# Parasomnia overlap disorder, Parkinson’s disease and subthalamic deep brain stimulation: three case reports

**DOI:** 10.1186/s12883-017-0916-0

**Published:** 2017-07-18

**Authors:** Panagiotis Bargiotas, Julia Muellner, W.M. Michael Schuepbach, Claudio L. Bassetti

**Affiliations:** Department of Neurology, University Hospital (Inselspital) and University of Bern, Freiburgstrasse 18, 3010 Bern, Switzerland

**Keywords:** Overlap parasomnia, Non-REM parasomnia, REM sleep behavior disorder, Sleep, Deep brain stimulation, Parkinson’s disease

## Abstract

**Background:**

Parasomnia overlap disorder (POD) is a distinct parasomnia and characterized by concomitant manifestation of rapid-eye-movement (REM)- and non-REM (NREM)-parasomnias. Although not uncommon among patients with Parkinson’s disease, POD is often under-investigated.

**Case presentation:**

This is the first report of patients with PD and features of POD that underwent deep brain stimulation. Our patients exhibited different outcomes of POD features after subthalamic deep brain stimulation.

**Conclusions:**

We expect that the reporting of these first patients will open the discussion about the need for more detailed and broad-spectrum assessments regarding parasomnias in PD patients that undergo deep brain stimulation. The implications of our observations are both clinical and neurobiological.

## Background

Parasomnia overlap disorder (POD) is a distinct parasomnia and characterized by concomitant manifestation of rapid-eye-movement (REM)- and non-REM (NREM)-parasomnias. There are only limited data on the prevalence of POD in the course of Parkinson’s disease (PD), and a previous questionnaire-based study reported that 26 out of 417 (6%) PD patients reported sleepwalking and also features of REM behavior disorder (RBD), suggesting the presence of POD. [1] To the best of our knowledge, this is the first report of PD patients with POD features in the context of subthalamic deep brain stimulation (STN-DBS).

## Methods

Standard video-polysomnography (v-PSG) was performed in all presented cases as previously described. [1] The v-PSG consisted of six channel EEG (F3/A2, F4/A1, C3/A2, C4/A1, O1/A2, O2/A1), left and right electrooculography, submental, left and right anterior tibialis, flexor carpi radialis and adductor digiti minimi electromyography, electrocardiography, respiratory flow and effort, and pulse-oximetry. All recordings were performed by Embla RemLogic™ Software. Sleep stages and sleep-associated events were manually scored according to the ICSD-III [2].

Levodopa equivalent daily dose (LEDD) was calculated as previously described (including all commonly used medications in PD patients such as dopamine agonists, MAO-B inhibitors, COMT-inhibitors etc.) [3].

## Case presentations

### Case 1

Patient 1, female, age 70 was diagnosed in 1999 with tremor-dominant PD, underwent bilateral STN-DBS in 2015. Since 2012, the quality of sleep gradually deteriorated mainly due to disrupted night sleep and early morning awakening resulting in daytime sleepiness (Epworth Sleepiness Scale, ESS:12/24). She reported weekly sleep-talking but no sleep-walking or dream-enacting behavior (DEB).

In v-PSG prior to STN-DBS, quantification of EMG-activity [4] showed increased phasic/tonic muscle activity and several episodes of DEB during REM-sleep. NREM-parasomnia included repeated mumbling, sleep-talking and confusional arousals.

STN-DBS was well received and 1 year after surgery, motor signs, assessed by Unified Parkinson’s Disease Rating Scale (UPDRS) part III, were ameliorated (57%) and LEDD was reduced by 91% (from 885 mg to 75 mg). Pregabaline 50 mg (prescribed due to very mild restless legs symptoms) and quetiapine 25 mg were gradually stopped after DBS surgery. The rest of medication (levothyroxine and painkillers) remained unchanged.

**Fig. 1:**
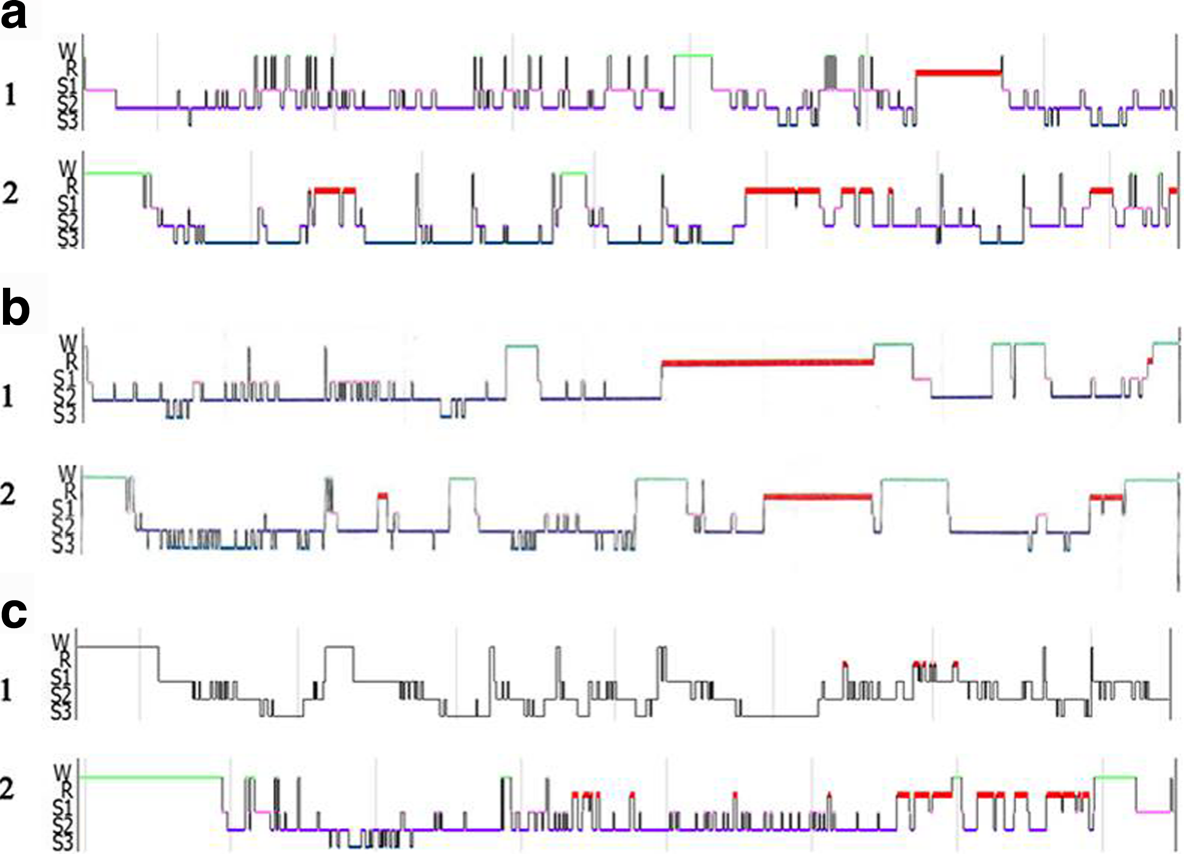
Polysomnography. **a** Hypnogram prior to surgery (1) showing a reduced sleep latency (1 min), low percentage of slow wave and REM sleep, increased NREM stage 1 sleep and arousal index of 33.3/h. One year after STN-DBS (2) sleep architecture improved with normal sleep latency (21 min), less frequent arousals (arousal index of 12.4/h), normal distribution of sleep stages and several REM-NREM cycles (Stimulation on). **b** Hypnogram prior to surgery (1) showing a reduced sleep latency (1.5 min), low percentage of slow wave NREM3 sleep (2.5%), one long-lasting and one very short-lasting phase of REM and normal NREM stage 1 sleep and arousal index of 33.9/h. One year after STN-DBS (2) sleep architecture improved slightly with normal sleep latency (17 min), less frequent arousals (arousal index of 8.6/h), normal distribution of sleep stages and 3 REM-NREM cycles (Stimulation on). **c** Hypnograms after STN-DBS showing the progression of the initially moderate disrupted sleep architecture in 2012 (1) into ambiguous sleep and status dissociatus in 2016 (2). In 2016, scoring of REM sleep was based on the presence of “saw tooth waves” in EEG and REM despite the absence of muscle atonia (Stimulation on). Time in hours: minutes on the x-axis. Sleep stages on y-axis: stage W (wake), stages S1-S3 (NREM), and stage R (REM)

Sleep quality and daytime sleepiness (ESS 5/24) improved and sleep-talking became rare. Sleep architecture improved markedly (Fig. 1, a1–2). Awakenings from stage 3 NREM were present but showed no episodes of DEB or confusional arousals. Compared to baseline, EMG showed a 44% reduction in tonic and 8% reduction in phasic activity. Apnea–Hypopnea Index (AHI) improved from 19.2/h to 9.2/h.

At the time of postoperative assessments stimulation parameters were:

STN (right): monopolar stimulation, C + 9-, 60 msec pulse width, 2.5 V amplitude and 125 Hz repetition / STN (left): interleave stimulation, a) C + 1-, 2.2 V (2.0–3.4 V) amplitude, 60 msec pulse width, 125 Hz repetition and b) C + 0-, 60 msec pulse width, 2.5 V amplitude, 125 Hz repetition.

### Case 2

Patient two, male, age 75, was diagnosed with rigid-hypokinetic PD in 2002, underwent bilateral STN-DBS in 2015. He reported refreshing sleep of good quality, no insomnia and only minor daytime sleepiness (ESS 7/24). His wife reported occasional episodes of sleep-walking, sleep-talking and DEB.

v-PSG prior to STN-DBS revealed increased phasic/tonic muscle activity [4] and several episodes of DEB during REM sleep as well as NREM-parasomnia including bruxismus, movements of all four extremities, sleep-talking, sounds of laughing/crying and grimacing several times throughout the night.

STN-DBS was well received and 1 year after surgery, motor signs, assessed by UPDRS part III, improved (50%) and LEDD was reduced by 70% (from 1500 mg to 460 mg). The rest of the medication (painkillers) remained unchanged. Sleepiness slightly improved (ESS 3/24), however, sleep quality deteriorated and his wife reported that frequency of sleepwalking and DEB doubled after DBS. In v-PSG, sleep architecture showed no significant changes (Fig. 1, b1–2). During REM sleep, while tonic activity was reduced (from 36 to 15% of total REM sleep), phasic activity increased (from 22 to 28%). During stage 3 NREM, episodes of confusional arousal were captured. AHI changed from 7/h to 2/h.

At the time of postoperative assessments stimulation parameters were:

STN (right): bipolar stimulation, 9–10+, 5.5 V (4.9–6.1 V) amplitude, 60 msec pulse width, 130 Hz repetition /STN (left): bipolar stimulation, 2–3+, 4.5 V (4.5–5.1 V) amplitude, 60 msec pulse width, 130 Hz repetition.

### Case 3

Patient three, male, age 55, diagnosed with tremor-dominant PD in 1994, underwent bilateral STN-DBS in 2003. Prior to STN-DBS, LEDD was 900 mg and sleep quality was poor (no sleep scores available).

After STN-DBS, motor signs improved (85% in UPDRS part III), sleep-wake functions remained unchanged and he remained free from dopaminergic medication until 2010 (at that time stimulation parameters were: STN (right): monopolar stimulation, C + 1-, 2.5Volt, 60 msec pulse width, and 145 Hz repetition/STN (left): monopolar stimulation, C + 1-, 3.5 V, 60 msec pulse width, 145 Hz repetition). Since 2010, LEDD was gradually increased from 0 mg to 560 mg (2013) and to 635 mg (2016) while the dose of venlafaxine 150 mg daily remained unchanged between 2010 and 2015 (2015 increased to a 300 mg dose). No anticholinergic drugs were given.

Since 2012, sleep quality progressively deteriorated, daytime sleepiness worsened (in 2012 ESS 07/24, in 2016 ESS 12/24), DEB frequency increased (previously only rare episodes were reported) and he developed prominent insomnia. Nocturnal behavior was characterized by sudden movements, unintelligible sounds and falling out of bed associated with vivid dreams and sleep-walking (2×/week).

v-PSGs in 2012 and 2014 illustrated a progressive increment in phasic/tonic muscle activity during REM sleep. NREM-parasomnia in 2012 included axial myoclonus, mumbling, sleep-talking, chewing, and spitting. In 2016, v-PSG showed a status dissociatus with constant movements of all extremities, sleep-talking, laughing, grimacing and tongue-clicking throughout the whole night (Fig. 1, c1–2). DEB-episodes coincided with “saw tooth waves” in EEG and REM, without associated muscle atonia. AHI gradually increased from normal in 2012 to 10/h in 2014 and 19/h in 2016.

At the time of 2016 postoperative assessments, stimulation parameters were:

STN (right): monopolar stimulation, C + 1-, 2.6 V amplitude, 60 msec pulse width, and 140 Hz repetition/STN (left): monopolar stimulation, C + 2-, 3.0 V, 90 msec pulse width, 130 Hz repetition.

## Discussion and Conclusions

DBS is a well-established treatment for motor symptoms in PD. Despite the impact of sleep disturbances on quality of life of patients with PD, [5] only few DBS studies included sleep and polysomnographic assessments prior to and after surgery (for review see Eugster et al. [6]). Considering sleep-wake functions as an integral part of the DBS outcome in PD patients, we report the first three PD patients with features of POD that underwent STN-DBS.

The pathological substrate of POD in PD remains unclear. Previous studies implicated neurodegeneration at the brainstem, possibly Lewy pathology, [7] involving the ascending control of state transition and the descending control of muscle tone. [8] At the neural level, gross motor phenomena during REM or NREM sleep may arise from a dissociated activity between serotonergic neurons that modulate tonic motor activity and those in the raphe nuclei regulating REM-NREM states, [9] implicating serotonergic system in the motor activity during REM and NREM sleep that characterizes POD. Changes in sleep quality and behavior after STN-DBS can be attributed to improved mobility or respiration and changes in medication. However, STN-related neuronal pathways may also directly be involved in sleep physiology mainly through reciprocal projections to structures involved in generation and modulation of sleep stages (including raphe nucleus, pedunculopontine nucleus and laterodorsal tegmentum), [10–12] and to regions that regulate circadian rhythms, mainly the thalamus [13, 14].

In the presented cases, it is difficult to disentangle the impact of STN-DBS, medication and disease progression itself on POD outcome. In cases 1 and 2, the very different course regarding outcome of POD features after STN-DBS underlies the important role of DBS itself or even differences in the stimulation parameters (e.g. bipolar vs monopolar stimulation) since in both cases there was a prominent LEDD reduction (90% and 70% respectively) and a comparable improvement in motor signs. In case 3, the alterations in sleep behavior, including deterioration of sleep quality, progressive development of POD and status dissociatus were observed only several years after STN-DBS and 2 years after readmission of dopaminergic therapy, underlying the important role of disease progression and possibly of the medication.

We expect that the reporting of these first patients will open the discussion about the need for more detailed and broad-spectrum assessments regarding parasomnias in PD patients that undergo DBS, with the goal of including these assessments in future prospective studies. This, not only will advance pre-DBS selection procedures, it will contribute to the improvement of postoperative therapeutic practice ﻿and may give new insights into the neurobiology of motor and sleep-wake control.

## Abbreviations

AHI: Apnea-hypopnea index
COMT: Catechol-O-Methyltransferase
DBS: Deep brain stimulation
DEB: Dream-enacting behavior
EEG: Electroencephalography
EMG: Electromyography
ESS: Epworth sleepiness scale
ICSD-III: The international classification of sleep disorders – third edition
LEDD: Levodopa equivalent daily dose
MAO: Monoaminooxidase
NREM: Non-rapid eye movement
PD: Parkinson’s disease
POD: Parasomnia overlap disorder
RBD: REM sleep behavior disorder
REM: Rapid eye movement
STN: Subthalamic nucleus
v-PSG: Video polysomnography

## References

[1] Di Fabio N, Poryazova R, Oberholzer M (2013). Sleepwalking, REM sleep behaviour disorder and overlap parasomnia in patients with Parkinson’s disease. Eur Neurol.

[2] American Academy of Sleep Medicine. International Classification of Sleep Disorders, 3rd ed. Darien: American Academy of Sleep Medicine; 2014.

[3] Tomlinson CL, Stowe R, Patel S (2010). Systematic review of levodopa dose equivalency reporting in Parkinson's disease. Mov Disord.

[4] Khalil A, Wright MA, Walker MC (2013). Loss of rapid eye movement sleep atonia in patients with REM sleep behavioral disorder, narcolepsy, and isolated loss of REM atonia. J Clin Sleep Med.

[5] Havlikova E, van Dijk JP, Nagyova I (2011). The impact of sleep and mood disorders on quality of life in Parkinson's disease patients. J Neurol.

[6] Eugster L, Bargiotas P, Bassetti CL, et al. Deep brain stimulation and sleep-wake functions in Parkinson's disease: A systematic review. Parkinsonism Relat Disord. 2016;32:12–9. 10.1016/j.parkreldis.2016.08.006 27605426

[7] Iranzo A, Tolosa E, Gelpi E (2013). Neurodegenerative disease status and post-mortem pathology in idiopathic rapid-eye-movement sleep behaviour disorder: an observational cohort study. Lancet Neurol.

[8] Poryazova R, Waldvogel D, Bassetti CL (2007). Sleepwalking in patients with Parkinson disease. Arch Neurol.

[9] Juszczak GR, Swiergiel AH (2005). Serotonergic hypothesis of sleepwalking. Med Hypotheses.

[10] Aravamuthan BR, Muthusamy KA, Stein JF (2007). Topography of cortical and subcortical connections of the human pedunculopontine and subthalamic nuclei. NeuroImage.

[11] Karimi M, Golchin N, Tabbal SD (2008). Subthalamic nucleus stimulation-induced regional blood flow responses correlate with improvement of motor signs in Parkinson disease. Brain.

[12] Lim AS, Moro E, Lozano AM (2009). Selective enhancement of rapid eye movement sleep by deep brain stimulation of the human pons. Ann Neurol.

[13] Magill PJ, Bolam JP, Bevan MD (2000). Relationship of activity in the subthalamic nucleus-globus pallidus network to cortical electroencephalogram. J Neurosci.

[14] Maquet P, Degueldre C, Delfiore G (1997). Functional neuroanatomy of human slow wave sleep. J Neurosci.

